# Comparative analysis of lipid components in fresh *Crassostrea Hongkongensis* (raw) and its dried products by using high-performance liquid chromatography/quadrupole time-of-flight mass spectrometry (HPLC/Q-TOF-MS)

**DOI:** 10.3389/fnut.2023.1123636

**Published:** 2023-03-10

**Authors:** Qunzhao Sun, Yunru Wang, Qiuxing Cai, Tingcai Pang, Weibing Lan, Laihao Li

**Affiliations:** ^1^Guangxi College and University Key Laboratory of High-Value Utilization of Seafood and Prepared Food in Beibu Gulf, College of Food Engineering, Beibu Gulf University, Qinzhou, China; ^2^College of Marine Science, Beibu Gulf University, Qinzhou, China; ^3^College of Light Industry and Food Engineering, Guangxi University, Nanning, China; ^4^Key Laboratory of Aquatic Product Processing, Ministry of Agriculture and Rural Affairs of the People's Republic of China, National R&D Center for Aquatic Product Processing, South China Sea Fisheries Research Institute, Chinese Academy of Fishery Sciences, Guangzhou, China

**Keywords:** *Crassostrea hongkongensis*, high performance liquid chromatography/quadrupole time-of-flight mass spectrometer, lipidomics, phosphoglyceride, multivariate statistical analysis

## Abstract

The lipids of the oyster (*Crassostrea hongkongensis*) have a special physiological activity function, which is essential to maintain human health. However, comprehensive research on their lipids species and metabolism is not so common. In our study, based on the high-performance liquid chromatography/quadrupole time-of-flight mass spectrometer (HPLC/Q-TOF-MS), the non-targeted lipidomics research of *Crassostrea hongkongensis* fresh and dried products was determined. Meanwhile, we analyzed its lipid outline, screened the differences between the lipid molecules of *Crassostrea hongkongensis* fresh and dried products, and determined the lipid metabolic pathway. Results showed that 1,523 lipid molecules were detected, in which polyunsaturated fatty acids mostly existed in such lipids as phosphoglyceride. Through the multivariate statistical analysis, according to the conditions of *P* < 0.05, FC > 2 or FC < 0.05, and VIP > 1.2, 239 different lipid molecules were selected, including 37 fatty acids (FA), 60 glycerol phospholipids (GP), 20 glycerin (GL), 38 sheath lipids (SP), 31 steroid lipids (ST), 36 polyethylene (PK), and 17 progesterone lipids (PR). Combined with the Kyoto Encyclopedia of Genes and Genomes (KEGG), the differential lipid molecules were analyzed to mainly determine the role of the glycerin phospholipid metabolic pathway. As a whole, the results of this study provide the theoretical basis for the high-value utilization of oysters and are helpful to the development of oysters' physiological activity functions and deep utilization.

## 1. Introduction

The ocean has extremely rich marine resources by virtue of its vast area. Moreover, it provides human beings with many active substances for drug-homologous foods. However, the development and utilization of marine resources are far from enough. In recent years, due to the development of technology, the industrialization and utilization of marine resources have also become a hotspot among the current research (1–3). There are varieties of oysters all over the world, such as *Crossostrea gigas, Crassostrea gigas angulate, Crassostrea hongkongensis, Ostrea rivularis Gould*, and *Crassostrea sikamea* (4). In China, oysters have a wide range of breeding and huge yields (5, 6). According to relevant statistics, the total output of oysters in China in 2021 reached 5.7962 million tons (7). Its breeding range from the north to the Yalu River, to Hainan Province in the south, and there are breeding in coastal areas. As one of the four major producing areas of Chinese oysters, Guangxi's oyster breeding is mainly concentrated on the coast of Beibu Gulf. Only Qinzhou has an annual output of 297,000 tons of oysters (7), among which, *Crassostrea hongkongensis* has a dominant position.

Regarding the record of the excellent pharmacological value of oysters, it has existed since ancient times. In *Shennong's Herbal Classic of Materia Medica*, oysters can prevent and cure wind-cold, strengthen the body, and extend the life of human beings. Many studies have proven that oysters are a kind of nutritional food, and their active substances have the effects of antibiosis, antioxidation, anti-inflammation, blood sugar lowering, blood pressure, liver protection, and skin whitening (8). At present, the research on oyster nutritional ingredients is mostly concentrated on the preparation of peptides and their application in trauma repair (9), the role of polysaccharides in disease prevention and control (10, 11), the distribution and existence form of minerals (12), and many more. Nonetheless, there are few studies on oyster lipid components.

Since Han and Gross (13) proposed the relevant concepts of lipidomics in 2003, lipids were found to play a crucial role in the fields of disease prevention and control. Over the years, an enormous amount of research has been conducted to determine that multi-unsaturated fatty acids that are rich in seafood, contribute to reducing cardiovascular and cerebrovascular diseases, and also have anti-inflammatory and anti-aging effects (14, 15). In addition, the lipids of some marine sources have a different positive nature (16). Existing studies have proved that the phospholipid type docosahexaenoic acid (DHA) and eicosapentaenoic acid (EPA) play a protective role in liver damage (17). Compared with other sources of DHA and EPA, they have higher biological utilization and better antioxidant stability (18). However, there is a current dearth of comprehensive analysis regarding the lipid composition and metabolism in oysters, most of which focus on seasonal changes in oyster lipids (19) and the study of oxidation of lipids under different conditions (20), owing to the fact that it may be restricted by the technical level. Traditional lipid measurements methods such as color measurement method, fluorescent method, gas chromatography (GC), and gas chromatography-mass spectrometry (GC-MS) (21), not only consume a long time, low sensitivity but even with certain interference factors. The liquid color spectrum series technology is widely used in the analysis of marine biolip-based components on account of its high volume and accuracy (22–24). Therefore, this experiment was based on the application of HPLC/Q-TOF-MS to study the lipids of *Crassostrea hongkongensis*. Additionally, due to the fact that the meat of fresh oysters is easy to be corrupted and cannot be stored and transported for a long time, many oysters are processed as dried products (25). The drying process conditions of marine products can accelerate the fragmentation, degradation, and oxidation of lipids, in particular, unsaturated fatty acids (26). Through the analysis of the nutritional components of *Crassostrea hongkongensis* fresh and dried products, it can be seen that after the procedure of processing *Crassostrea hongkongensis*, the content of crude fat has changed significantly (27). Therefore, our study needed to determine the lipid molecules of *Crassostrea hongkongensis* fresh and dried products, and according to the composition and relative content of the lipid molecules of *Crassostrea hongkongensis* fresh and dried products, selecting different lipid molecules after the processing process of *Crassostrea hongkongensis* fresh, which clarified the changes in lipid molecules in the procedure of dry processing in *Crassostrea hongkongensis*. The resulting data would provide a better theoretical basis for the high-value utilization of oysters while helping the in-depth development of active substances and customizing the production processes to increase the health-promoting lipid content.

## 2. Materials and methods

### 2.1. Preparation of *Crassostrea hongkongensis* samples

The experiment purchased fresh shell *Crassostrea hongkongensis* from Dongfeng Market in Qinzhou, Guangxi Province, China. The oysters purchased were produced in Beibu Gulf. The specifications of oysters are *Crassostrea hongkongensis* (8 heads/100 g).

After purchasing, they were quickly transported back to the laboratory for the next process. The purchased shell *Crassostrea hongkongensis* samples were randomly divided into two parts, each of which weighed 500 g. Some of them were immediately homogenated and mashed as a sample, and −18°C freezes for subsequent experimental analysis. The rest of them need to go through an electro-thermostatic water bath. After cooking them for 3 min at 70°C, we continue to use an electric thermostatic drying oven to dry them at 70°C until the water content drops to 18 ± 1%, making them into dried oyster products. All tissue samples were separately pulverized using a stainless-steel mortar and pestle and mixed on an ultraclean bench and placed in a sterile food-grade plastic bag until further analysis.

### 2.2. Total lipid extraction

The two-part oysters were divided into four copies, in which Group A was *Crassostrea hongkongensis* fresh, and Group C was *Crassostrea hongkongensis* dried products. Take 50 mg of the four copies of *Crassostrea hongkongensis* fresh (Group A) and four copies of *Crassostrea hongkongensis* dried products (Group C) to the grinding pipe and add a steel ball with a diameter of 2 mm. Then, the samples were reconstituted in 100 μl extracting solution (2:5, V/V = methanol: water), and add 400 μl Methyl tert-Butyl Ether. Keep the temperature at −20°C, adjust the frequency to 50 Hz, and grind them with Tissuelyser-24 (Shanghai Jingxin Industrial Development Co., Ltd) for 6 min. Then set the temperature to 5°C, adjust the frequency to 49 kHz, and extract 30 min at a low-temperature ultrasound. Set the sample at −20°C for 30 min. The constitution was then centrifuged at 12,000 rpm for 15 min at 4°C, and 40 μl of supernatant was transferred to an EP tube and blown dry with nitrogen. Add 100 μl extracting solution (1: 1, V/V = isopropanol: acetonitrile). Sonicated them for 10 min in the ice-water bath at 5°C and 40 KHz. The constitution was then centrifuged at 12,000 rpm for 15 min at 4°C. Take the supernatant as a sample. Finally, mix the equal amount as a quality control (QC) sample.

### 2.3. HPLC/Q-TOF-MS analysis conditions

This study conducted a quantitative analysis of the lipids of *Crassostrea hongkongensis* fresh and dried products through HPLC/Q-TOF-MS technology. The HPLC separation was carried out using an Agilent 1,290 series HPLC System (NYSE: A, USA). The experiment utilized the 100^*^2.1 mm of the Acquity UPLC Beh C18 (Waters, USA), and the filler particles are 1.7 μm BEH particles. The mobile phase A consisted of a water solution with 0.1% formic acid in it. The mobile phase C consisted of 10% acetonitrile and 90% isopropanol formate. The column temperature was 45°C. The two groups of experiments were maintained at the column temperature of 40°C, the flow rate was 0.4 ml/min, and the injection volume was 4 μl.

Gradient program of the mobile phase: 0–2 min, 30–60% B; 2–8 min, 60–85% B; 8–10 min, 85–98% B; 10–15 min, 98% B; 15–16 min, 98–30% B; 16–20 min 30% B.

Q-TOF-MS is used to experiment with Agilent 6,545 Q-TOF (NYSE: A, USA), set the capillary voltage to 4,500 V, control the drying air temperature at 325°C and turn on the dry gas at a flow rate of 10 L/min. Keep the spray pressure at 20 PSIG, set the craft of the breaker to 120 V, and finally, collect the mass spectrometry data in the 200–2,000 m/z range for subsequent research and analysis.

### 2.4. Data analysis

In this experiment, the MSCONVERT software (Proteowizard) was employed to convert the detected ion peak and time signal to a general (MZXML) format. Progenesis QI software (Non-linear Dynamics, Newcastle, UK) was used for peak recognition, points, reserved time correction, and peak pairs, and then output the original mass spectrum peak area and finally normalize the data. The appraisal of the compound is based on accurate quality and secondary fragments and then uses the LipidMaps database for qualitative. Multiple statistics were analyzed using the R software platform and SIMCA-P 14.1.

## 3. Result and discussion

### 3.1. Lipid group learning detection quality control

**Figure 1:**
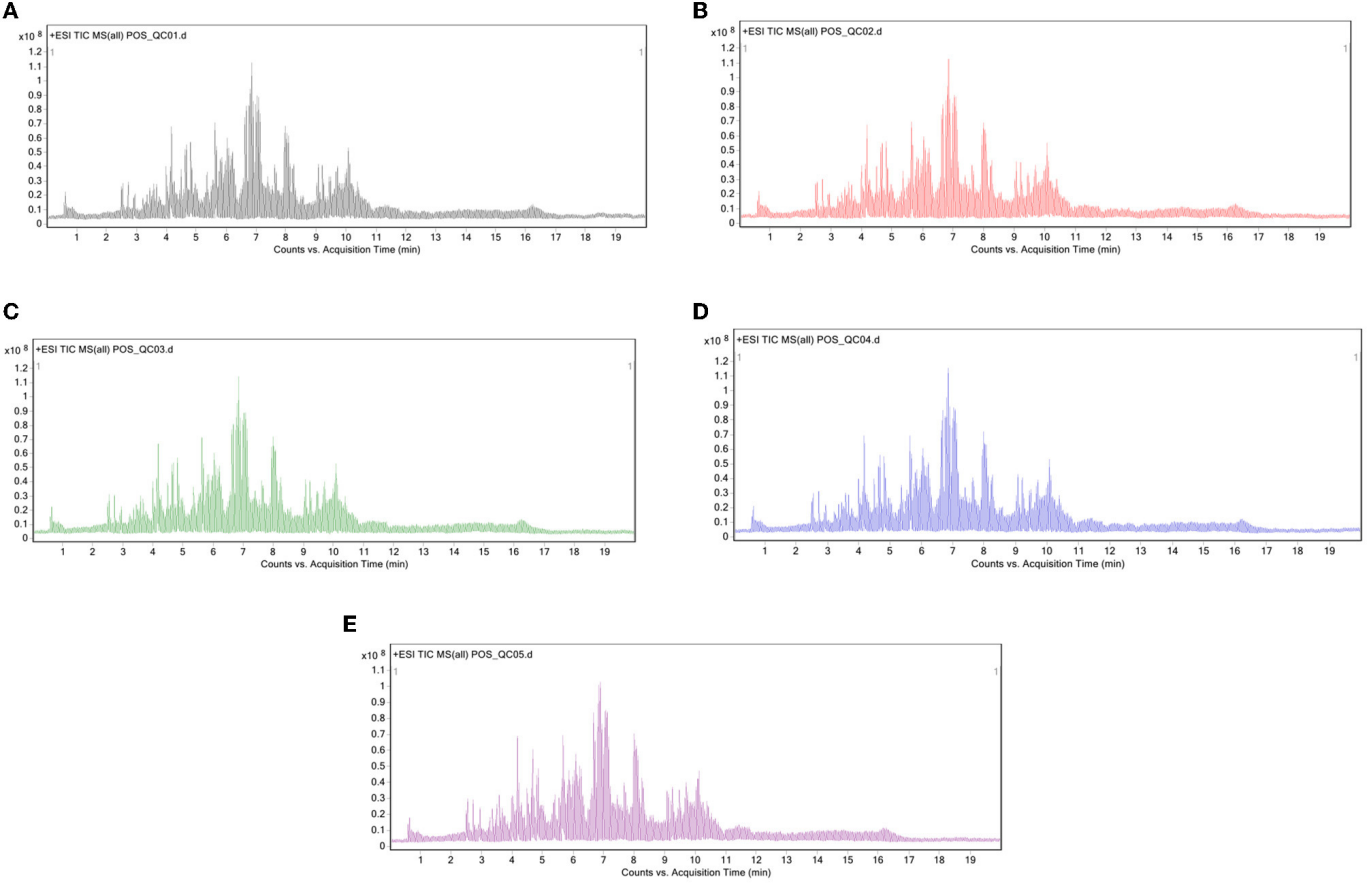
Total ion flow diagram of QC samples in positive ion mode: **(A–E)** First detection, second detection, third detection, fourth detection, and fifth detection.

The sample for this study consisted of *Crassostrea hongkongensis* fresh and dried products. Repeat QC samples five times to get samples and the total ion flow diagram (TIC) as shown in Figures 1A–E. QC samples had high reproducibility of TIC medium-color peaks, and the reserved time was compared with the time. This showed that the system stability of the instrument used in this study was high, and the data obtained was highly reliable, which can further analyze the lipid data measured by samples of *Crassostrea hongkongensis* fresh and dried products.

### 3.2. Lipid profile of *Crassostrea hongkongensis* fresh and dried products

**Figure 2:**
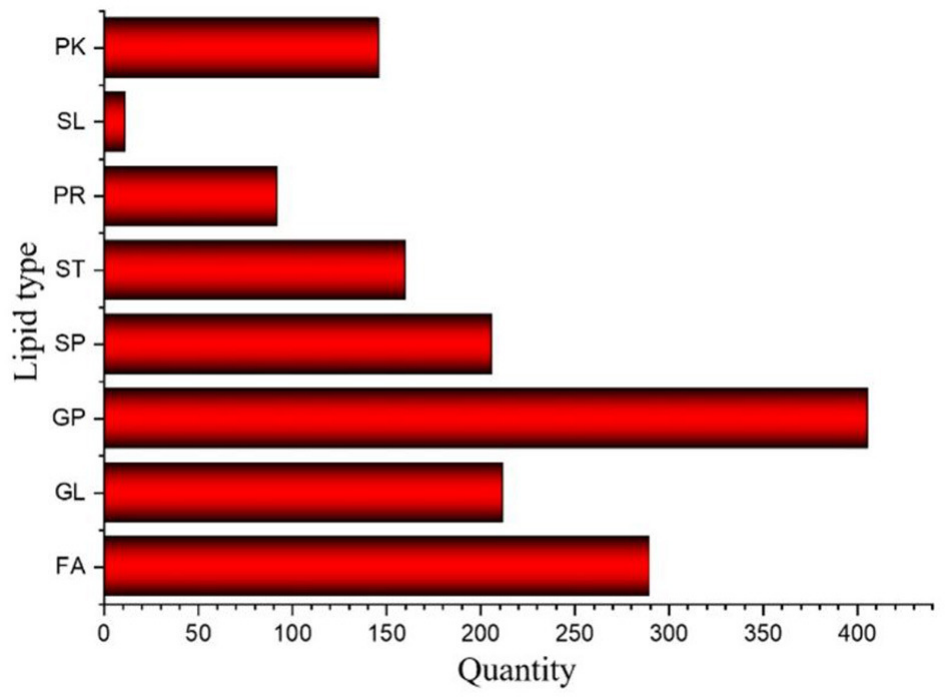
Number of lipid molecules in *Crassostrea hongkongensis* fresh and dried products.

**Figure 3:**
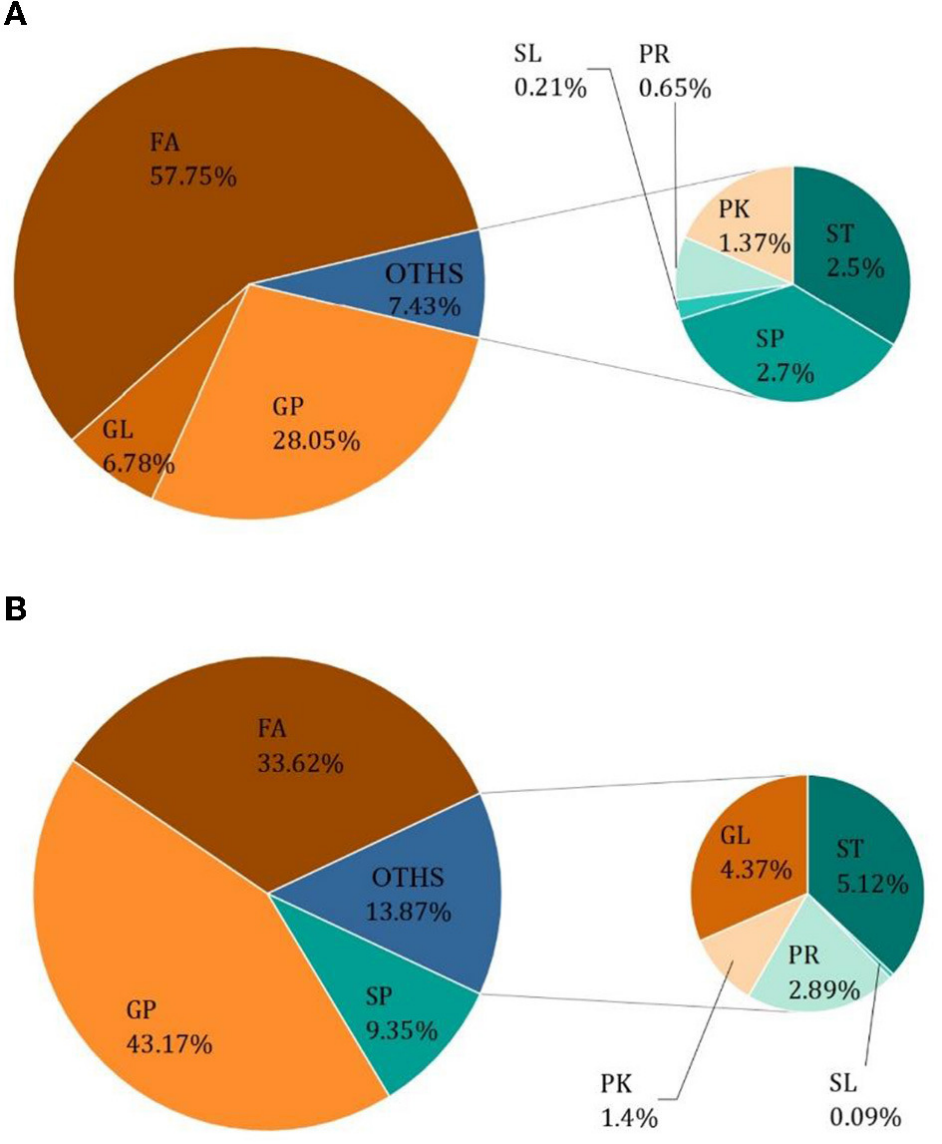
**(A)** Relative content of each lipid species in *Crassostrea hongkongensis* fresh products. **(B)** Relative content of each lipid species in *Crassostrea hongkongensis* dried products.

**Figure 4:**
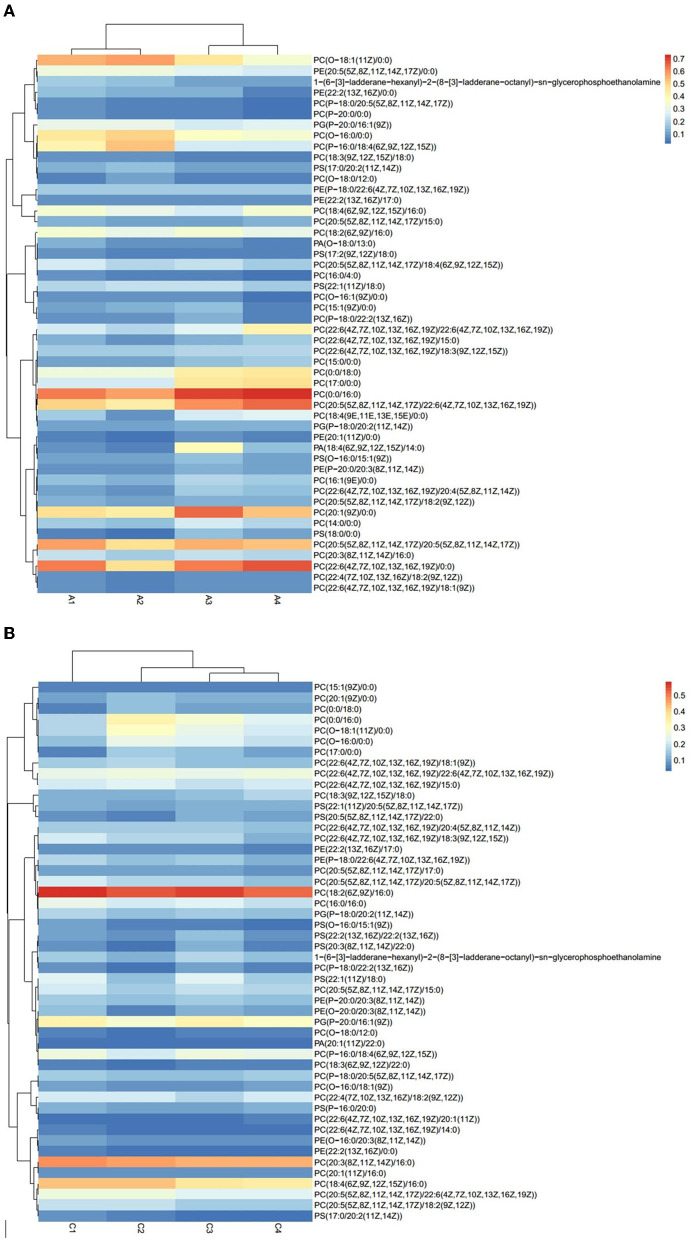
**(A)** Heat map of GP molecular content of *Crassostrea hongkongensis* fresh products. **(B)** Heat map of GP molecular content of *Crassostrea hongkongensis* dried products.

The results of *Crassostrea hongkongensis* fresh and dried products samples were filtered using the FilterVariable function to retain 25–75% of the sorted positions to obtain the final lipid molecules of *Crassostrea hongkongensis* fresh and dried products. The lipidomic analysis of *Crassostrea hongkongensis* fresh and dried products was based on HPLC/Q-TOF-MS. The lipids were classified according to their chemical structure and biosynthetic pathways into fatty acids (FA), glycerolipids (GL), glycerophospholipids (GP), sphingolipids (SP), sterol lipids (ST), pregnenolipids (PR), saccharolipids (SL), and polyketides (PK) (13). The lipid molecules measured for these eight classes of lipids were counted and are shown in Figure 2. A total of 1,523 kinds of lipid molecules were detected, including 290 FA, 212 GL, 406 GP, 206 SP, 160 ST, 92 PR, 11 SL, and 146 PK. This is a comprehensive representation of the lipid molecules in *Crassostrea hongkongensis* fresh and dried products. The relative content of each liposuction in *Crassostrea hongkongensis* fresh and dried products is shown in Figures 3A, B. Among them, the content of lipid molecules in *Crassostrea hongkongensis* fresh and dried products (Select the top 50 lipid molecules with a relative content from high to low) is detailed in Figure 3, where GP molecules are shown in Figures 4A, B. The relative content of each lipid category in *Crassostrea hongkongensis* fresh products is FA (57.75%), GP (28.05%), GL (6.87%), SP (2.70%), ST (2.50%), PK (1.37%), PK (1.37%) PR (0.65%), and SL (0.21%). As shown in Figure 2C, the relative content of each lipid category in *Crassostrea hongkongensis* dried products is FA (33.62%), GP (43.17%), GL (4.37%), SP (9.35%), ST (5.12%), PK (1.40%), PR (2.89%), and SL (0.09%). Most of the multi-unsaturated fatty acids exist in GP.

### 3.3. Multivariate statistical analysis of lipid species in *Crassostrea hongkongensis* fresh and dried products

#### 3.3.1. Principal component analysis

**Figure 5:**
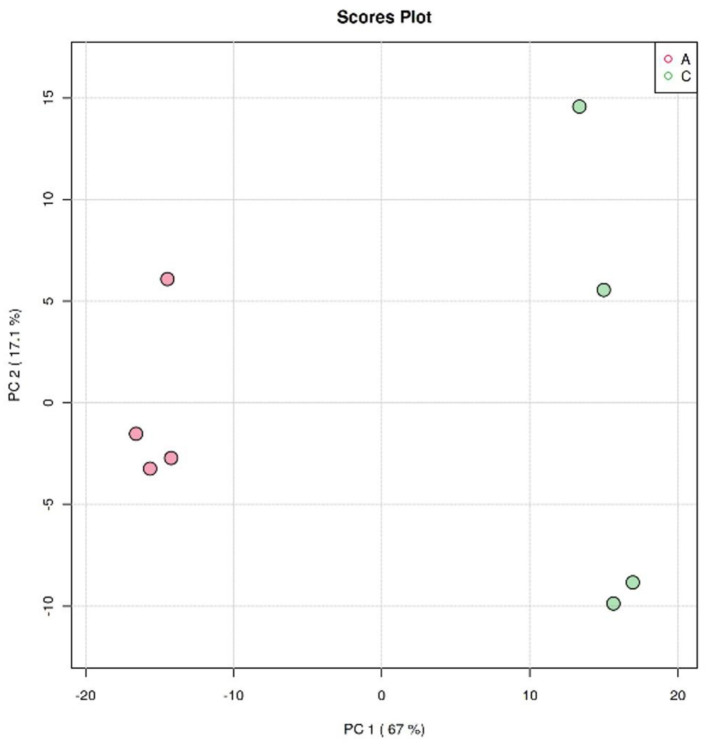
PCA score of *Crassostrea hongkongensis* fresh and dried products.

The clustering analysis of *Crassostrea hongkongensis* fresh and dried products was performed by the principal component analysis (PCA) model in unsupervised mode to observe the separation of *Crassostrea hongkongensis* fresh and dried products and to establish a lipidomic model. The score plot based on the PCA model is shown in Figure 5. PC1 and PC2 were the two principal components contributing the most to the model, with 67 and 17.1%, respectively, and the cumulative contribution of the principal components reached 84.1%, which could reflect most of the information of the original variable indicators in a comprehensive manner. The distribution of samples within the group of *Crassostrea hongkongensis* fresh products (Group A) was more concentrated, and the samples within the group of *Crassostrea hongkongensis* dried products (Group C) were more dispersed, but the separation between the groups of Group A and Group C was obvious, indicating that the characteristic lipid molecules were significantly different between Group A and Group C. The above results indicated that there were significant differences in lipid molecules between the *Crassostrea hongkongensis* fresh and dried products, and the drying process had a significant effect on the lipid molecules of the *Crassostrea hongkongensis* fresh products. Due to the dispersion of the samples within the group, it was necessary to consider the differences in lipid molecules within the group; hence, further analysis was performed using multivariate statistical methods in a supervised mode.

#### 3.3.2. Orthogonal partial least squares discriminate analysis

**Figure 6:**
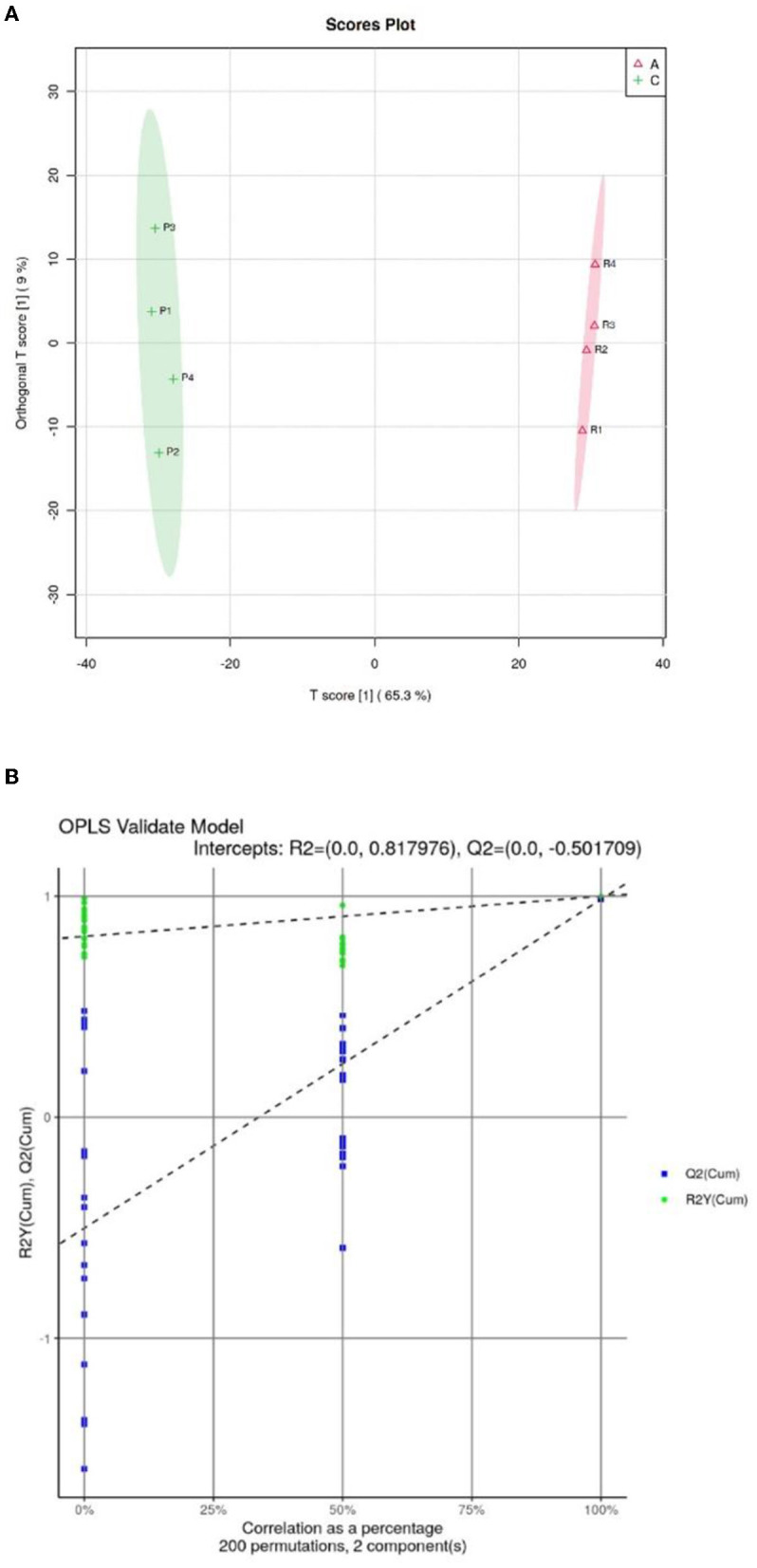
OPSL-DA score of *Crassostrea hongkongensis* fresh and dried products and replacement test of OPLS-DA model. **(A)** OPSL-DA score of *Crassostrea hongkongensis* fresh and dried products. **(B)** Replacement test of OPLS-DA model of *Crassostrea hongkongensis* fresh and dried products.

Orthogonal partial least squares discriminate analysis (OPSL-DA) based on a supervised model was used to model the lipidomics of the *Crassostrea hongkongensis* fresh and dried products. The OPLS-DA score diagram is shown in Figure 6A. The *Crassostrea hongkongensis* fresh product group (Group A) and the *Crassostrea hongkongensis* dried product group (Group C) were within the 95% confidence interval, respectively, and the two groups were completely separated with a large difference in the horizontal coordinates, indicating that the lipid molecules between the *Crassostrea hongkongensis* fresh and dried product were highly differentiated and the lipid molecules in them changed significantly after drying of the *Crassostrea hongkongensis* fresh product. The random model R2 and Q2 values and RY and Q regression lines were obtained by the replacement test (200 times) to determine whether the OPLS-DA model was over-fitted, and the results of the replacement test are shown in Figure 6B. The results of the permutation test are shown in Figure 6B. The R2 and Q2 of the stochastic model were lower than the original values of the model, and the intercept of the model Q2 regression line was −0.502, which was <0. This indicates that the OPLS-DA model established in this study is not over-fitted. In summary, according to the results of the permutation test, the stability and reliability of the OPLS-DA model established for *Crassostrea hongkongensis* fresh and dried products were high, with no over-fitting phenomenon, and the subsequent analysis of the relevant data was statistically significant.

### 3.4. Comparative and analysis of lipid differences between *Crassostrea hongkongensis* fresh and dried products

#### 3.4.1. Differential lipid screening of Crassostrea hongkongensis fresh products and dry products

**Figure 7:**
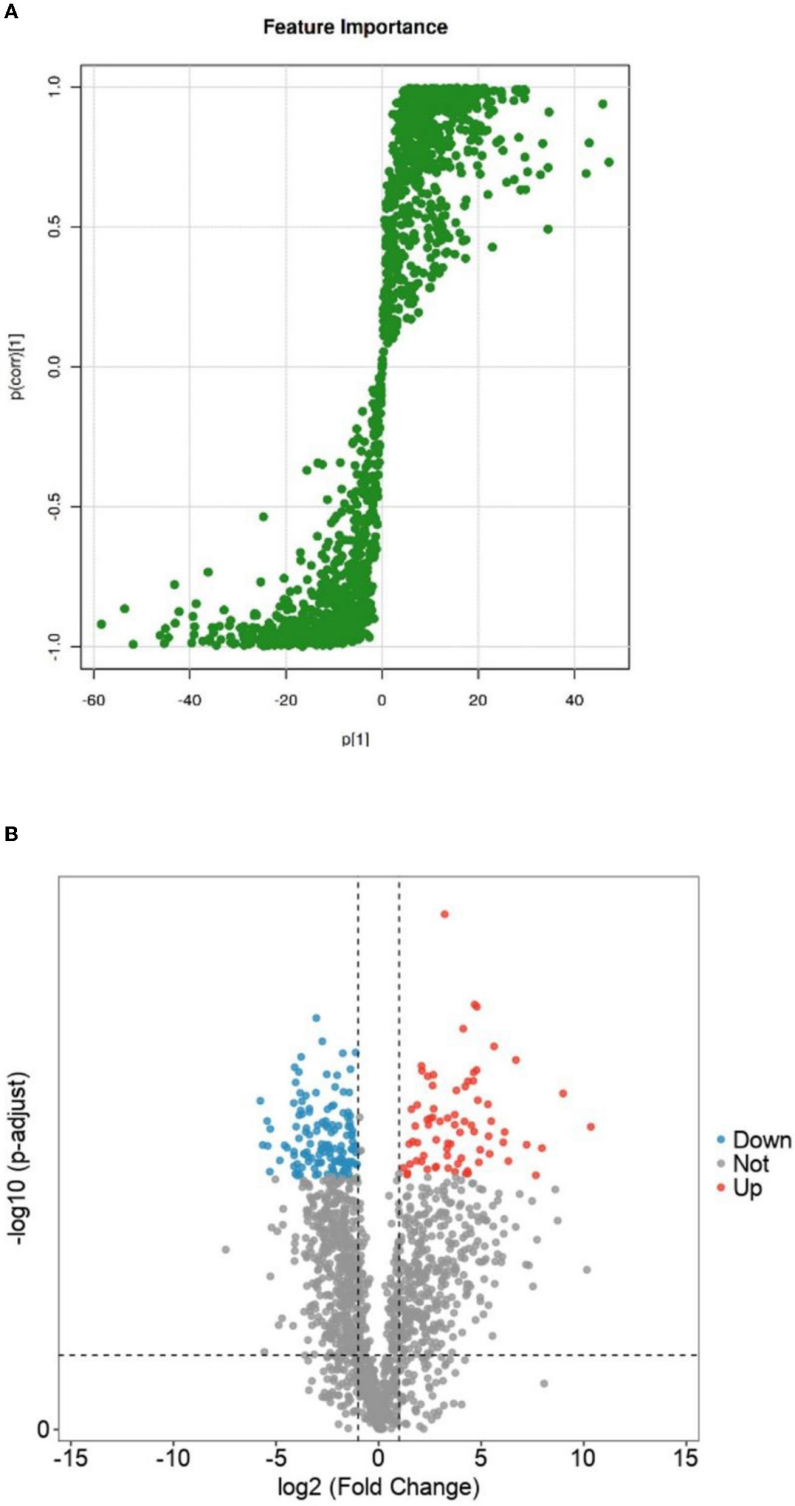
S-plot loadings of fresh and dried *Crassostrea hongkongensiss* and volcanoes of differential lipid molecule screening. **(A)** S-plot loadings of fresh and dried *Crassostrea hongkongensiss*. **(B)** Volcanoes of differential lipid molecule screening of fresh and dried *Crassostrea hongkongensiss*. In the figure, the red dot (Up) represented a lipid molecule that significantly increased *Crassostrea hongkongensiss* fresh and dried products; the blue dot (Down) represented a lipid molecule that was significantly reduced between the fresh content between *Crassostrea hongkongensiss* fresh and dried products; the gray dot (Not) indicated that there was no significant change in lipid compounds between *Crassostrea hongkongensiss* fresh and the dried products.

The OPSL-DA model outputted the S-PLOT load chart based on Section 3.3.2, as shown in Figure 7A. The S-PLOT load graph could identify statistically significant and potential biomarkers (28). The farther away from the scattered point of the origin or the scattering point at the edge area is, the more different the corresponding lipid molecules will be in *Crassostrea hongkongensis* fresh and dried products. It is a characteristic lipid molecule for changing *Crassostrea hongkongensis* fresh and dried products.

To further clarify the differential lipid molecules in *Crassostrea hongkongensis* fresh and dried products, they were screened according to the conditions of *P* < 0.05, FC > 2, or FC <0.5 by combining the *P*-value and Fold Change (FC) in the statistical method *T*-test and presented in the form of volcano plots. As shown in Figure 7B, there were 860 differential lipid molecules between the fresh and dried Hong Kong oyster products, in which 374 lipid molecules were significantly upregulated and 486 were significantly downregulated.

#### 3.4.2. Identification of differential lipid molecules

The number of differential lipid molecules between *Crassostrea hongkongensiss* fresh and dried products screened according to the *P* and FC values was large. In order to further screen for more significant differential lipid molecules, the screening was carried out by combining the variable importance in the projection (VIP) and using VIP > 1.2 as the screening condition. A total of 239 differential lipid molecules were screened, including 37 FA, 60 GP, 20 GL, 38 SP, 31 ST, 36 PK, and 17 PR. Meanwhile, according to the lipid subclasses, it could be found that the content of glycerol phosphate and neutrophils increased, while the glycerol phosphate and alcohol decreased (Table 1).

**Table 1:** Molecular classification of differential lipids in fresh and dried products of *Crassostrea hongkongensis*.

**Lipid category**	**Subcategory**	**Upregulate**	**Downregulate**
FA	Fatty acids and conjugates	7	8
	Octadecanoids	2	0
	Eicosanoids	0	3
	Fatty esters	2	2
	Fatty amides	3	8
	Fatty acyl glycosides	0	2
GP	Glycerophosphocholines	10	16
	Glycerophosphoethanolamines	2	6
	Glycerophosphoserines	2	4
	Glycerophosphoglycerols	1	2
	Glycerophosphoinositols	3	5
	Glycerophosphates	3	6
GL	Diradylglycerols	1	6
	Triradylglycerols	4	9
SP	Sphingoid bases	6	4
	Ceramides	1	0
	Phosphosphingolipids	0	3
	Neutral glycosphingolipids	14	2
	Acidic glycosphingolipids	5	3
ST	Sterols	6	13
	Steroids	0	1
	Secosteroids	5	4
	Bile acids and derivatives	1	0
	Steroid conjugates	1	0
PK	Flavonoids	2	34
PR	Isoprenoids	6	8
	Quinones and hydroquinones	1	2

### 3.5. Enrichment analysis of differential lipid molecule metabolic pathways

**Figure 8:**
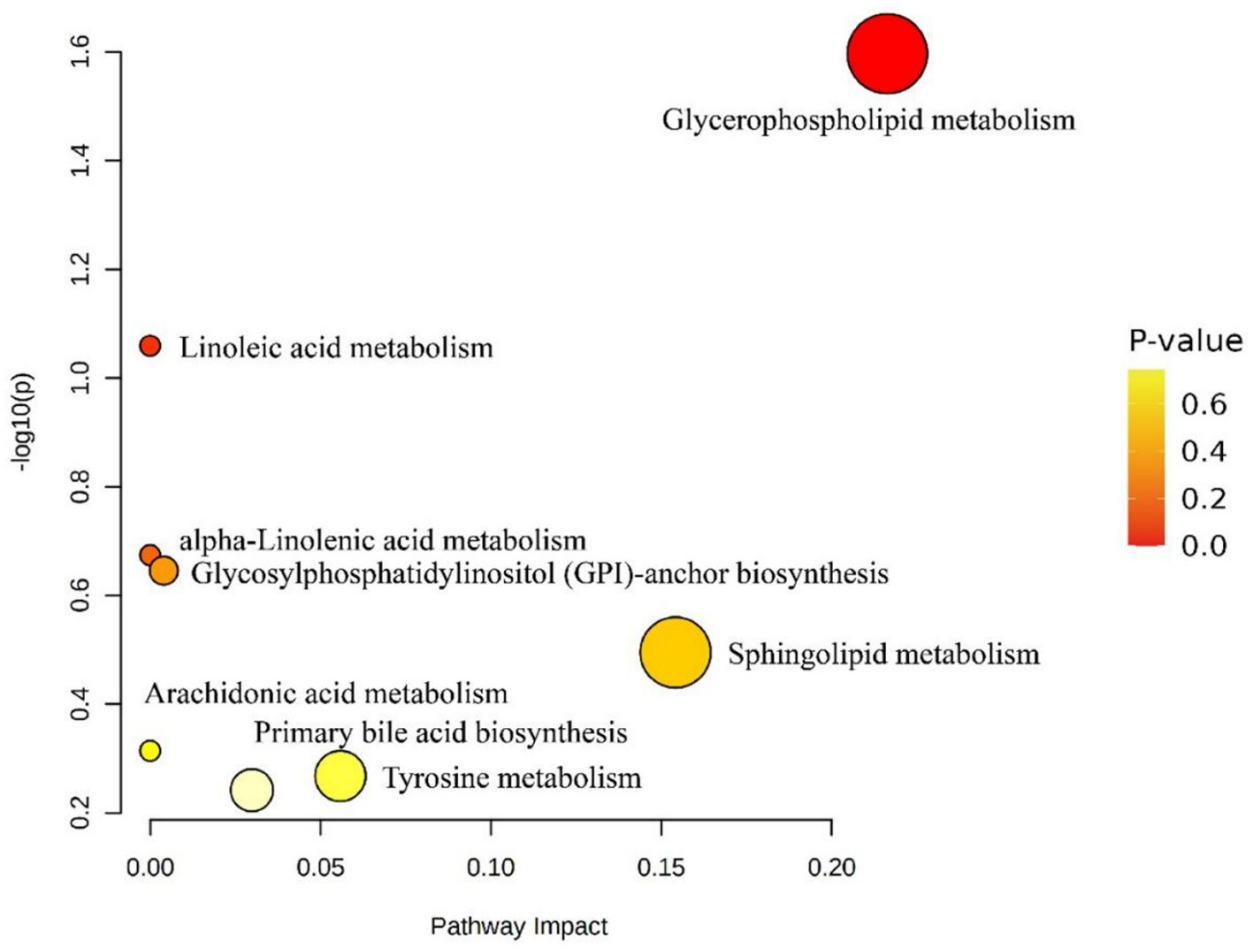
Effect of differential lipid molecule metabolic pathways in fresh and dried products of *Crassostrea hongkongensis*. Each bubble in the figure represented a metabolic channel, and its horizontal coordination and bubbles shall jointly indicate the influence of the corresponding metabolic pathway in the different lipid molecules of fresh and dried products of *Crassostrea hongkongensis*.

The screened lipid molecules with KEGG numbers were matched with the KEGG database through the Metabolomics Pathway Analysis software, and the results could be seen as shown in Figure 8. According to the conditions of *P* < 0.05, the matching pathway was screened to obtain the glycemic phospholipid metabolic pathway.

## 4. Discussion

Lipidomics is a system analysis of the molecularity of various lipids and interactions in organisms. The current lipidomics mainly includes lipids and their metabolites (29, 30), lipid function and metabolic regulation (31, 32), lipid metabolic channels and networks (33, 34), lipid biomarkers (35), and other research content. Lipidomics can be divided into targeted lipidomics and non-targeted lipidomics, depending on the requirements of the study. Targeted lipidomics characterizes known lipids and allows for multiple analyses, including absolute qualitative and quantitative analysis of the lipids to be measured using standards but has the disadvantage of having a limited number of lipids to measure; non-targeted lipidomics aims to capture all the lipids that can be measured, with the disadvantage that false-positive signals may interfere with the results and the data measured cannot be quantified absolutely (36). Li et al. (37) used UPLC-Q-Exactive Orbitrap Mass Spectrometry to analyze the lipidomics of goat, soy, and cow milk. It identified 14 kinds of lipids as biomarkers and established the PLS model, comparing three types of milk lipids. Sun et al. (38) used UPLC-QTOF-MS to perform untargeted lipidomics on hazelnut oil extracted by pressing, ultrasound-assisted hexane, and enzyme-assisted methods; measured 98 lipid molecules; and quantified the measured lipid molecules by combining stable isotope labeled internal standards. In this study, the lipids of Hong Kong oyster fresh and dried products were analyzed based on non-targeted lipidomics, aiming to identify the differences in lipid molecules present in *Crassostrea hongkongensis* fresh and dried products, and to further illustrate the dynamic changes in lipid profiles that occur in *Crassostrea hongkongensis* fresh products during the drying process.

This study determined the lipid molecules in positive ion mode, filtered through the Filter variable function, and only retained the results of 25–75% of the sorting position. A total of 1,523 lipid molecules were measured and 406 kinds of phospholipids in it. Among the common foods in daily life, milk as a non-aquatic product contains 378 kinds of lipid molecules (39), and Gadus as a member of an aquatic product contains 498 kinds of lipid molecules (40). By contrast, the types of lipid molecules of *Crassostrea hongkongensis* are more abundant.

Among them, polyunsaturated fatty acids of *Crassostrea hongkongensis* mostly exist in GP. Among the relative content of each lipid category in *Crassostrea hongkongensis*, the relative content of non-esterified fatty acid (57.75%) was the highest, followed by GP (28.05%), and glycerin lipids (6.87%). Furthermore, there are rich types of phospholipids, with more than 160 phospholipid molecules owned by *Apostichopus japonicus* (41). In *Crassostrea hongkongensis* dried products, the relative content of fatty acids (33.62%) decreased, the relative content of GP (43.17%) increased substantially, and the relative content of glycerin lipid (4.37%) decreased. Previous studies showed that Neri et al. (42) and Luo et al. (43) during the hot air drying process, the lipid components were oxidized violently. Additionally, the oxidation rate of fatty acids was fast, followed by triglycerides. These data also demonstrated our study, that is, during the process of drying, the content of its lipid composition has changed significantly. It has been shown that the fresh products of *Crassostrea hongkongensis* have higher nutritional value compared to their dried products. In addition, studies have shown the positive effect of omega-3 polyunsaturated fatty acids in fish oil on human health (44). Based on the anti-cardiovascular activity of unsaturated fatty acids as described previously (45), as well as the characteristics of low-free fatty acid content and low glycerin content, *Crassostrea hongkongensis* can be used as a good healthy food. At the same time, the study of specific molecular types of oysters can be used for further research and applied to nutrition and medicine.

Studies have shown that PC and PE are important phospholipids, and they are also important parts of glycerin phospholipid metabolism (46). Our results suggested that in GP, the relative content of phospholipidized choline was significantly reduced, and the content of hemolytic phospholipidized alkaline was significantly increased. The reason was that under the action of enzymes, phosphatidylcholine produced hemolytic phospholipidal alkaline through hydrolysis (47), which was consistent with the glycerin phospholipid metabolic pathway by KEGG.

In this study, the effects of seasonal changes of *Crassostrea hongkongensis* and cultural environmental problems on the lipid composition of *Crassostrea hongkongensis* were not emphasized, and no other methods were used to process *Crassostrea hongkongensis* dried products and conducted component analysis and discussion. However, in future research, it is necessary to comprehensively understand and analyze the above. Furthermore, research should further study new nutritional functions. Just as recent studies have shown Sun et al. (48), the study of lipidomics can provide a basis for a reasonable diet and further clarify the fat composed of specific composition, such as the relationship between PUFAs and related metabolic diseases. Exploring the digestion and absorption process of oyster lipids on the human body and the biological synthetic pathway of oyster phospholipid. In short, the results of our research are capable of providing the theoretical basis for the high-value utilization of oysters and a certain reference for the development of oysters' physiological activity functions and deep utilization.

## 5. Conclusion

To summarize, comprehensive lipid information including qualitative and quantitative results in *Crassostrea hongkongensis* fresh and dried products were determined by using HPLC/Q-TOF-MS. The results showed that dehydration reduced the relative content of fatty acids and increased the relative content of GP in *Crassostrea hongkongensis* fresh and dried products. In addition, multivariate statistical analysis was adopted to screen a total of 239 different lipid molecules. Among them, the up-regulate lipid molecules existed more in glycerol phosphate and neutral sheathose lipids, and the down-regulate lipid molecules were more in glycerine phosphate and sterol. By combining the KEGG database to match the metabolic path analysis, the results showed that the different lipid metabolic pathways in *Crassostrea hongkongensis* fresh and dried products were mainly through glycerin phospholipid pathways. The number of lipid molecular species that could be detected and the analysis of lipids in common oysters were quite rare in previous studies. These massive data provided a more sophisticated and well-equipped basis for further research, such as the high-value utilization of oysters and the mechanism of phospholipids on health.

## Data availability statement

Publicly available datasets were analyzed in this study. This data can be found here: www.ebi.ac.uk/metabolights/MTBLS6719.

## Author contributions

QS and YW: writing—original draft, data curation, and conceptualization. QC: writing—review and editing and conceptualization. TP and WL: visualization. LL: project administration. All authors contributed to the article and approved the submitted version.
